# Developments of Conventional and Microfluidic Flow Cytometry Enabling High-Throughput Characterization of Single Cells

**DOI:** 10.3390/bios12070443

**Published:** 2022-06-23

**Authors:** Minruihong Wang, Hongyan Liang, Xiao Chen, Deyong Chen, Junbo Wang, Yuan Zhang, Jian Chen

**Affiliations:** 1 State Key Laboratory of Transducer Technology, Aerospace Information Research Institute, Chinese Academy of Sciences, Beijing 100190, China; wangminruihong19@mails.ucas.ac.cn (M.W.); lianghongyan17@mails.ucas.ac.cn (H.L.); chenxiao20@mails.ucas.ac.cn (X.C.); dychen@mail.ie.ac.cn (D.C.); 2 School of Future Technology, University of Chinese Academy of Sciences, Beijing 100049, China; 3 School of Electronic, Electrical and Communication Engineering, University of Chinese Academy of Sciences, Beijing 100049, China; 4 Materials Genome Institute, Shanghai University, Shanghai 200444, China

**Keywords:** single-cell analysis, optoelectronic flow cytometry, hematology analyzer, microfluidic impedance and imaging flow cytometry

## Abstract

This article first reviews scientific meanings of single-cell analysis by highlighting two key scientific problems: landscape reconstruction of cellular identities during dynamic immune processes and mechanisms of tumor origin and evolution. Secondly, the article reviews clinical demands of single-cell analysis, which are complete blood counting enabled by optoelectronic flow cytometry and diagnosis of hematologic malignancies enabled by multicolor fluorescent flow cytometry. Then, this article focuses on the developments of optoelectronic flow cytometry for the complete blood counting by comparing conventional counterparts of hematology analyzers (e.g., DxH 900 of Beckman Coulter, XN-1000 of Sysmex, ADVIA 2120i of Siemens, and CELL-DYN Ruby of Abbott) and microfluidic counterparts (e.g., microfluidic impedance and imaging flow cytometry). Future directions of optoelectronic flow cytometry are indicated where intrinsic rather than dependent biophysical parameters of blood cells must be measured, and they can replace blood smears as the gold standard of blood analysis in the near future.

## 1. Introduction

Single-cell analysis is aimed to qualitatively and/or quantitatively estimate biological heterogeneities across cell populations to describe their physiological and/or pathological stages. The explorations of cell heterogeneity (e.g., the dynamics of how heterogeneity arises in a cell population) can be leveraged to build theoretical models of predicting dynamics of healthy populations (e.g., immune variation) and understanding signal pathways to determine disease progresses (e.g., tumor heterogeneity) [1,2,3].

As for tools and instruments of Single-cell analysis, they can be mainly classified into approaches in Single-cell genotyping and Single-cell phenotyping. In the field of Single-cell genotyping, a variety of instruments have been developed to realize Single-cell sequencing, while big-data information at the Single-cell level can be obtained to explore two key scientific problems: landscape reconstruction of cellular identities during dynamic immune processes [4,5,6] and mechanisms of tumor origin and evolution [7,8,9]. This portion was discussed in detail in Section 2 Scientific Meaning of Single-cell Analysis.

In the area of Single-cell phenotyping, flow cytometry has been functioning as the gold standard instrument in high-throughput quantification of biophysical and/or biochemical properties of single cells. More specifically, label-free optoelectronic flow cytometry measures electrical impedance and/or optical scattering of travelling single cells, which is heavily demanded in the clinical needs of complete blood counting [10,11,12]. Meanwhile, multicolor fluorescent flow cytometry measures fluorescent intensities of travelling single cells with multiple antigens labeled with fluorescence conjugated antibodies, which is heavily demanded in the clinical needs of diagnosing hematologic malignancies [13,14,15]. This portion was discussed in detail in Section 3 Clinical Demands of Single-cell Analysis.

In order to better clarify the clinical demands of complete blood counting, historical developments of optoelectronic flow cytometry were then included, where 3-part differential, 5-part differential and 5-part + differential of leukocytes were realized in a time sequence [16]. In addition, the performances of the state-of-art optoelectronic flow cytometry (hematology analyzers) in complete blood counting were presented and compared. Although these well-established optoelectronic flow cytometries can be used to realize 5-part + differential of leukocytes, they cannot be used to classify immature white blood cells and thus low-throughput and labor-intensive microscopic smears are still required when abnormal leukocytes are encountered in whole blood counting. This portion was discussed in detail in Section 4 Well-Established Optoelectronic Flow Cytometry.

Driven by the trend of miniaturization, microfluidic optoelectronic flow cytometry has been developed where geometrical parameters of sensitive structures can be fine-tuned, leading to increases in detection accuracies. In this article, key technical developments in microfluidic impedance [17,18] and imaging [19,20] flow cytometry were covered and their applications in leukocyte characterization were highlighted. For detailed information, please refer to Section 5 Microfluidic Optoelectronic Flow Cytometry for Characterizing Individual Blood Cells. Future directions of developing next-generation microfluidic optoelectronic flow cytometry enabling the characterization of Single-cell intrinsic biophysical parameters to form commonly used quantitative identification systems of leukocytes were also included in this article at the Section 6.

## 2. Scientific Meaning of Single-Cell Analysis

The immune system is a heterogeneous system consisting of a variety of cell types working in a well-coordinated manner to defend invading pathogens and protect tissue damages. Thus, Single-cell analysis plays key roles in this area by realizing high resolutions and throughputs of phenotyping individual immune cells within networks. However, reconstructing landscapes of cellular identities during dynamic immune processes both in differentiation and antigenic responses remains an elusive method due to limited functions of current tools of Single-cell analysis [4,5,6] (see Box 1).

In solid tumors, the tumor is a “heterogeneous system” that includes benign cells, malignant cells exhibiting varied genetic information and a variety of stromal cells (e.g., immune cells, fibroblasts and vasculature cells), which play key roles in tumor developments, progression, metastasis, and responses to therapies. Currently, tools of Single-cell analysis, such as Single-cell sequencing, have contributed to identifications of genetic mutations within tumors, pathway understanding of signaling and metabolism, and optimization of treatment regimens. However, due to low throughputs of these analytical tools at the Single-cell level, complete answers remain elusive for two key scientific problems in tumor heterogeneity which are mechanisms of tumor origin and evolution, and varied responses to tumor therapies [7,8,9] (see Box 1).

Box 1 **Scientific problems in the field of single-cell analysis.**

**Immune Variation:**

**Meaning:**

The immune system is a heterogeneous system consisting of a variety of immune cell types and works in a well-coordinated manner to defend invading pathogens and protect tissue damages.

**Problem:**

Landscape reconstruction of cellular identities during immune dynamic processes in differentiation and antigenic responses.

**Tumor Heterogeneity:**

**Meaning:**

The tumor is a heterogeneous system that includes benign cells, malignant cells exhibiting varied genetic information and a variety of stromal cells which collaboratively contribute to tumor developments.

**Problem:**

Exploration of mechanisms of tumor origin and evolution, and varied responses to tumor therapies.

## 3. Clinical Demands of Single-Cell Analysis

As for clinical demands of Single-cell analysis, the main method comprises a complete blood count enabled by optoelectronic flow cytometry and diagnosis of hematologic malignancies enabled by multicolor fluorescent flow cytometry, which are described as follows (see Box 2).

In a complete blood count, which is a well-established clinical examination, optoelectronic flow cytometry (hematology analyzer, a key instrument of Single-cell analysis) is heavily demanded, which measures electrical impedance and/or optical scattering of individual blood cells without labeling of antibodies. As the first clinical test, a complete blood count provides key insights about cellular components of the blood, which are red blood cells of RBC (anemia vs. erythrocytosis), white blood cells of WBC (leukopenia vs. leukocytosis) and platelets of PLT (thrombocytopenia vs. thrombocytosis). In addition, levels of leukocyte differential provide rich information regarding likely types and causes of underlying diseases (e.g., infectious, inflammatory or neoplastic). Thus, the complete blood count functions as the first indicator of disease and plays as a pivotal starting point in forming a clinical diagnosis and for monitoring disease progression or treatments [10,11,12].

In the diagnosis of hematologic malignancies, multicolor fluorescent flow cytometry (a key instrument of Single-cell analysis) is heavily demanded, which measures fluorescent intensities of individual leukocytes with multiple antigens labeled with fluorescence conjugated antibodies. In a common practice of hematologic malignancy, the existence of atypical lymphocytes or immature blast cells requires the analysis of multicolor fluorescent flow cytometry with panels of leukemia and lymphoma. This malignancy is first classified into myeloblasts or lymphoblast, which is then grouped into an erythroid, monocytic, lymphoid or undifferentiated lineage. After the diagnosis based on the multicolor fluorescent flow cytometry, further pathological tests can be conducted to determine a detailed differential which contributes to a specific treatment [13,14,15]. Note that in comparison to optoelectronic flow cytometry, multicolor fluorescent flow cytometry is featured with a higher specificity and thus used for the classification of subtypes of malignant leukocytes with myeloid or lymphoid lineages. Meanwhile, compared to optoelectronic flow cytometry, multicolor fluorescent flow cytometry suffers from the issue of higher cost due to the use of antibodies and thus it is not commonly used in whole blood counting.

Box 2 **Clinical demands in the field of Single-cell analysis.**

**Complete Blood Count:**

**Meaning:**

In a complete blood count, enumeration of blood cells provides key insights on anemia vs. erythrocytosis, leukopenia vs. leukocytosis, thrombocytopenia vs. thrombocytosis, while WBC differentials provide rich information on infectious, inflammatory or neoplastic.

**Instrument:**

As a key instrument of Single-cell analysis, label-free optoelectronic flow cytometry (hematology analyzer) is heavily demanded, which measures electrical impedance and/or optical scattering of individual leukocytes without labeling.

**Diagnosis of Hematologic Malignancies:**

**Meaning:**

In a typical case of hematologic malignancy, it is first classified into myeloblasts or lymphoblast, which is further differentiated into a monocytic, megakaryocytic, erythroid, B/T-lymphoid or undifferentiated lineage.

**Instrument:**

As a key instrument of single-cell analysis, multicolor fluorescent flow cytometry is heavily demanded, which measures fluorescent intensities of individual leukocytes with multiple antigens labeled with fluorescence conjugated antibodies.

## 4. Well-Established Optoelectronic Flow Cytometry (Hematology Analyzer)

### 4.1. Historical Development

As the first-type instrument of Single-cell analysis, optoelectronic flow cytometry (hematology analyzer) with key historical events are briefly reviewed in this study as follows (see Table 1) [16].

In 1953, W. Coulter patented the first “hematology analyzer” which became commercially available as Model A of Coulter Electronics. In this prototyping instrument, a cell travelled through an aperture between two electrodes, producing an electric pulse due to insulating properties of cell membrane. In 1977, an automatic hematology analyzer (Model S Plus) was developed by Coulter Electronics where a three-part differential of WBC (a large group of neutrophils or NEU, an intermediate group of monocytes or MONO and a small group of lymphocytes or LYM) was realized based on variations of sizes and corresponding resistance. 

As for hematology analyzers of realizing five-part differentials of WBC (e.g., NEU, MONO, LYM, eosinophils or EOS, basophils or BASO), Model STKs (Coulter Electronics) collected 22 parameters with a throughput of 100 samples per hour, where DC/AC impedance and optical scattering were used to measure cell sizes, physical and chemical structures and compositions. Sysmex NE-8000 from TOA Medical Electronics measured 23 parameters with a processing speed of 120 sample/hour, where both direct currents and radio frequencies were leveraged for size estimation and extraction of nuclear parameters. Note that in this instrument, separated channels were included to enumerate EOS and BASO based on corresponding chemical treatments and size measurements. Additionally, Cell-DYN 3000 of Abbott reported 22 parameters using multiple-angle light scattering to capture sizes, granularities, and internal complexities of individual WBC.

### 4.2. DxH 900 of Beckman Coulter

**Figure 1:**
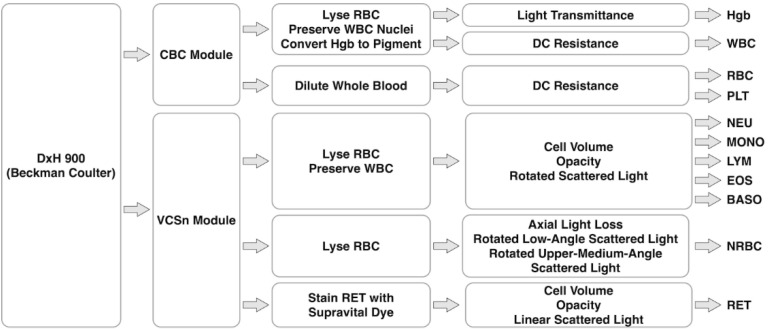
Working flowcharts of DxH 900 (Beckman Coulter), which is mainly composed of a CBC module for complete blood counting based on DC resistance, and a VCSn module for 5-part differential of WBC, NRBC and RET based on cell volume, opacity and scattered light.

The DxH 900 Analyzer is the state-of-art hematology analyzer of Beckman Coulter, which is capable of a complete blood counting (e.g., RBC, WBC and PLT) based on a CBC module and a 5-part and more differential (NEU, MONO, LYM, EOS, BASO, nucleated red blood cells or NRBC, reticulocytes or RET) based on a VCSn module at a throughput of ~100 samples per hour (see Figure 1).

In the CBC module composed of RBC and WBC triple aperture baths, a portion of the whole blood is diluted to ~6000 folds for the counting of RBC and PLT based on the Coulter Principle while another portion of the whole blood is diluted and lysed to a volume of ~250 times, which is flushed into the aperture both for counting WBC. Then, the lysed WBC dilution is measured at the light transmittance at 525 nm to determine the concentration of the hemoglobin (Hgb). 

Meanwhile, all Diff, NRBC, and RET analysis occurs in the VCSn module, which includes three measurements of cell volume by low-frequency current, high-frequency conductivity and multiple angles of light scatter. More specifically, the five-part differential is realized by cell volume, impedance ratio of high and low frequency (opacity) and rotated light scatter. The differentiation of NRBC from WBC is enabled by axial light loss, rotated low-angle light scatter and rotated upper-medium-angle light scatter. Furthermore, differentiation of RET from RBC and PLT is based on cell volume, linear light scatter, and opacity where staining of supravital dyes is conducted to precipitate and aggregate basophilic substances within RET to form a granular pattern. 

### 4.3. XN-1000 of Sysmex

**Figure 2:**
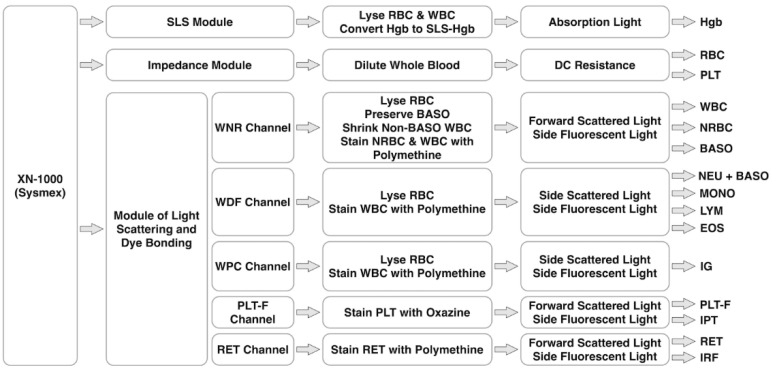
Working flowchart of XN-1000 (Sysmex), which is mainly composed of a SLS module for Hgb detection based on absorption light, an impedance module for RBC and PLT counting based on DC resistance, and a module of light scattering and dye bonding for 5-part differential of WBC, NRBC, IG, PLT-F, IPT, RET and IRF based on scattered and fluorescent lights.

The XN-1000 Analyzer is the state-of-art hematology analyzer of Sysmex, which is capable of counting RBC and PLT based on an impedance module with a hydrodynamic focusing and a 5-part and more differential (NEU, MONO, LYM, EOS, BASO, NRBC, RET, immature granulocyte of IG, immature reticulocyte fraction of IRF and immature platelet fraction of IPF) based on light scattering and dye bonding at a throughput of ~100 samples per hour (see Figure 2).

For the module of light scattering and dye bonding, forward scattered light (FSL), side scattered light (SSC) and side fluorescent light (SFL) of a stained cell excited with a laser beam of 633 nm are measured to determine cell size, intracellular information of cell organelles and nucleic acids.

More specifically, in the WNR channel, the whole blood is treated with a special low-pH reagent to maintain BASO and shrink other WBC and lyse the membrane of RBC and specifically label the nucleus of NRBC. Then, FSC and SFL are used to classify NRBC, BASO and non-BASO WBC. In the WDF channel, RBC are lysed and WBC are stained fluorescently based on polymethine dyes, and then SSC and SFL are used to classify NEU+BASO, MONO, LYM and EOS.

In the WPC channel, RBC are lysed, and immature and mature WBC are fluorescently stained differentially by polymethine dyes due to variations in lipid compositions of cell membranes, and then SSC and SFL are used to classify immature, abnormal and mature WBC. In the PLT-F channel of specifically staining PLT based on oxazine dyes, FSC and SFL are used to classify PLT and immature platelet fraction of IPF. In the RET channel with nuclear staining of polymethine dyes, FSC and SFL are used to classify RBC, RET and immature reticulocyte fraction of IRF.

### 4.4. ADVIA 2120i of Siemens

**Figure 3:**
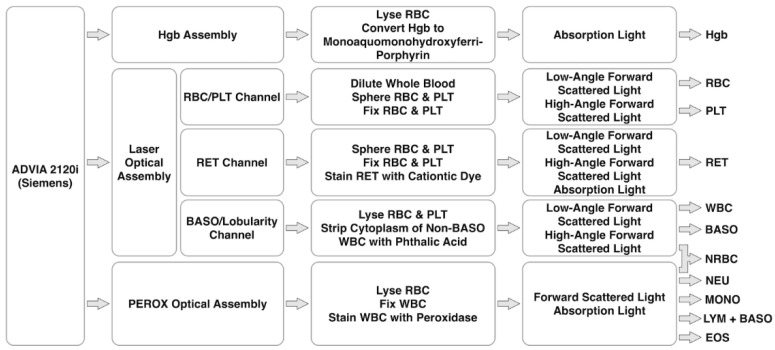
Working flowchart of ADVIA 2120i (Siemens), which is mainly composed of a Hgb assembly for Hgb detection based on absorption light, a laser optical assembly for complete blood counting, RET and BASO based on scattered and absorption lights, and a PEROX optical assembly for 5-part differential of WBC based on scattered and absorption lights.

The ADVIA 2120i Analyzer is the state-of-art hematology analyzer of Siemens, which is capable of complete blood counting, white cell differential counts and reticulocyte absolute at a throughput of ~120 samples per hour (see Figure 3).

In the laser optical assembly, a laser diode light source is used to differentiate RBC and PLT, RET, and BASO/Lobularity with light absorption, a low-angle scatter signal between 2° and 3° and a high-angle scatter signal between 5° and 15°. Within the channel of BASO/Lobularity, WBC except BASO are stripped of their cytoplasm and can be classified as mono/polynuclear cells leveraging geometrical information of their nuclei while the undamaged BASO can be simply discriminated from smaller nuclei.

Furthermore, within the channel of RBC and PLT, they are sphered by sodium dodecyl sulfate and fixed by glutaraldehyde, which are then classified based on the pair of low-angle and high-angle light scatters. In the channel of RET, RET are stained differently according to varied contents of nucleic acids, producing higher intensities of light absorption than mature RBC.

In the chamber of PEROX reaction, followed by RBC lysis, WBC are fixed and then stained with 4-Chloro-1-naphthol which serves as substrates that enable the hydrogen peroxide to precipitate within the granules of WBC with peroxidase activities. NEU, MONO and EOS are labelled due to their high levels of peroxidase activities while LYM and BASO remain unlabeled since they contain low expressions of peroxidase. In the PEROX optical assembly, scattering between 5° and 10° and absorption over a 0° and 10° angular interval of a tungsten light beam are measured, leading to the differentiation of a mixture of NEU, MONO, LYM + BASO and EOS. 

As for the counting of NRBC, in the stain-free domain of the Peroxidase channel, nuclei of NRBC are found between LYM and the noise, forming distinct countable populations. In the BASO/Lobularity channel, nuclei of NRBC are located in the polymorphonuclear domain since they are with higher densities than nuclei of MONO or LYM. 

### 4.5. CELL-DYN Ruby of Abbott

**Figure 4:**
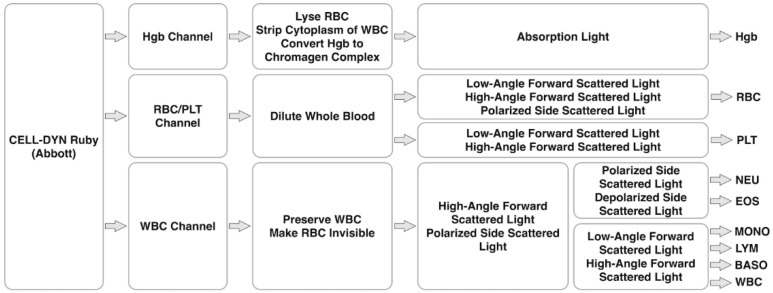
Working flowchart of CELL-DYN Ruby (Abbott), which is mainly composed of a Hgb channel for Hgb detection based on absorption light, an RBC/PLT channel for REC and PLT counting based on scattered light, and a WBC channel for 5-part differential of WBC based on scattered light.

As a key hematology analyzer of Abbott, the CELL-DYN Ruby Analyzer utilizes techniques of flow cytometric to count RBC, PLT and WBC at a throughput of 84 samples per hour (see Figure 4). In this hematology analyzer, a technique of multi-angle polarized scatter separation is included where scattered light is captured at the forward angles of 0° and 10° and side angles of 90° and 90° D.

For the classification of WBC, forward scatter at 10° and side scatter at 90° are used to form clusters of polymorphonuclear and mononuclear cells. Then, polymorphonuclear cells are further classified into NEU and EOS based on side scatters at 90° and 90° D while mononuclear cells are further classified into MONO, LYM and BASO based on forward scatters at 0° and 10°. In the channel of RBC/PLT, the scattered light is captured at 90°, 10° and 0° for counting RBC and at 10° and 0° for counting PLT.

## 5. Microfluidic Optoelectronic Flow Cytometry for Characterizing Individual Blood Cells

### 5.1. Microfluidic Impedance Flow Cytometry

In microfluidic impedance flow cytometry, individual cells travel rapidly throughput a microfabricated detection region with fine-tuned electric fields and the corresponding impedance variations are captured for cell-type classification and cell-status evaluation [17,18]. In comparison to conventional optoelectronic flow cytometry, microfluidic counterparts can accurately define detection geometries and electric fields, leading to increases in detection accuracy for Single-cell impedance analysis (see Table 2).

**Figure 5:**
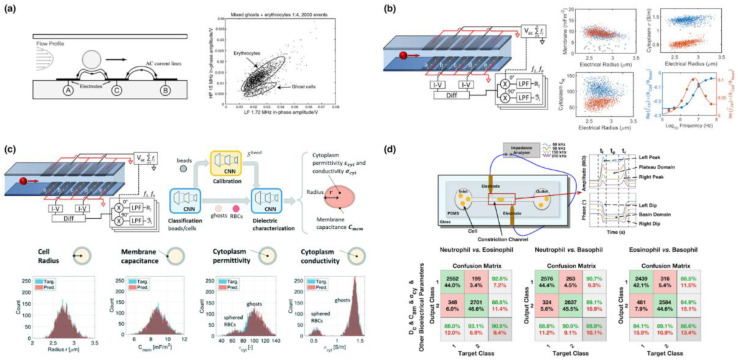
Key developments of microfluidic impedance flow cytometry, (**a**) coplanar microelectrodes for differentiation of healthy and ghost RBC based on AC impedance [21]; (**b**,**c**) parallel microelectrodes for differentiation of healthy and ghost RBC based on intrinsic bioelectrical properties of single cells enabled by Maxwell’s mixture theory [34] and convolutional neural network [38]; (**d**) constriction microchannels for five-part differential of white blood cells based on both AC impedance and intrinsic bioelectrical properties of single cells [37]. Figures were reprinted with permissions from (**a**) Royal Society of Chemistry, copyright 2001; (**b**) American Chemical Society, copyright 2020; (**c**) Royal Society of Chemistry, copyright 2022 and (**d**) John Wiley and Sons, copyright 2022.

As pioneers in this field, in 2001, Renaud@EPFL firstly reported microfluidic impedance flow cytometry based on coplanar microelectrodes where two-frequency impedance variations due to travelling blood cells were captured. The low-frequency impedance data were used to estimate cell diameters while the high-frequency impedance data were used to characterize internal portions of individual cells, leading to the differentiation of healthy and ghost RBCs [21] (see Figure 5a).

In 2009, based on parallel microelectrodes of microfluidic impedance flow cytometry, Morgan@Southampton realized the three-part differential of white blood cells leveraging two-frequency impedance values [23]. Compared to the coplanar microelectrodes, the impedance flow cytometry based on parallel microelectrodes can obtain a higher difference of impedance with and without a travelling cell. Furthermore, leveraging Maxwell’s mixture theory [34] and convolutional neural network [38], Morgan et al. translated preliminary impedance profiles into intrinsic bioelectrical markers such as specific membrane capacitance and cytoplasmic conductivity, enabling the classification of healthy and ghost RBCs (see Figure 5b,c).

Meanwhile, Chen@CAS reported the microfluidic impedance flow cytometry based on constriction microchannels where, based on an equivalent circuit model for a cell squeezing through the microchannel, impedance profiles were translated into intrinsic bioelectrical markers of cell diameter, specific membrane capacitance and cytoplasmic conductivity [25]. Based on these intrinsic bioelectrical markers, three-part differential of white blood cells were realized by the same group [36]. Furthermore, by integrating preliminary impedance profiles and intrinsic bioelectrical markers of single cells travelling through the constriction microchannels, five-part differential of white blood cells were also reported by Chen@CAS [37] (see Figure 5d).

### 5.2. Microfluidic Imaging Flow Cytometry

In microfluidic imaging flow cytometry, individual cells travel rapidly throughout a microfabricated detection region with fine-tuned geometrical properties and the corresponding morphology variations are captured for cell-type classification and cell-status evaluation [19,20]. In comparison to conventional optoelectronic flow cytometry, microfluidic counterparts can accurately define positions of travelling single cells and geometries of optical fields, leading to increases in detection accuracy for Single-cell imaging analysis.

**Figure 6:**
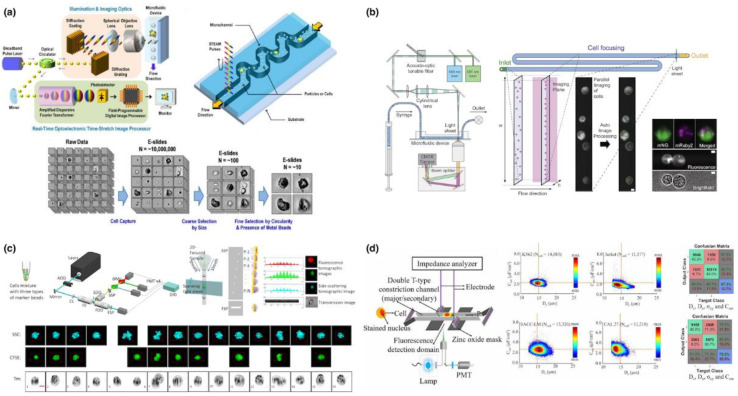
Key developments of microfluidic imaging flow cytometry, (**a**) inertial focusing for differentiation of MCF-7 vs. WBC [24]; (**b**) viscoelastic focusing for imaging yeast and 293T [35]; (**c**) spatial filter with microfabricated slits and pinholes for differentiation of tumor cells [39]; (**d**) constriction microchannel with microfabricated window for differentiation of tumor cells [40]. Figures were reprinted with permissions from (**a**) Proceedings of the National Academy Sciences, copyright 2012; (**b**) copyright 2021, the author(s); (**c**) copyright 2022, the author(s) and (**d**) copyright 2022, the author(s).

From the perspective of defining positions of travelling single cells, Goda@UCLA in 2012 developed a microfluidic imaging flow cytometry leveraging inertial focusing and a serial time-encoded photodetector [24]. In this study, without sandwiching fluid flows in conventional flow cytometry, inertial microfluidics was adopted to confine cells within detection planes of optical lens, enabling the differentiation of rare MCF-7 and WBC at a throughput of 100,000 cells/s (see Figure 6a). However, in inertial focusing, for a travelling cell, there were multiple equilibrium positions within microfluidic channels, leading to the issue of out of focus and thus compromised imaging qualities of single cells.

In order to address this issue, in 2021, deMello@ETH reported a microfluidic imaging flow cytometry incorporating viscoelastic focusing and scientific CMOS. In this study, due to the nature of viscoelastic focusing traveling cells were confined to the center of the microfluidic channels within focus planes of optical elements, and then dual-color fluorescence and bright filed images were captured by the scientific CMOS at a throughput of 60,000 cells/s for yeast and 293T cells [35] (see Figure 6b). However, this approach still suffered from the key limitations of out of focus since different from inertial focusing, the accurate position of travelling cells by viscoelastic focusing can be heavily affected by multiple factors including physical properties of solutions used to suspend cells, channel and cell geometries and cell travelling velocities.

From the perspective of defining geometries of optical fields, in 2015, Lo@UCSD reported a microfluidic imaging flow cytometry based on microfabricated slits where time signals obtained by a PMT can be translated to geometrical information of travelling cells. This approach was reported to image A549 cells at a throughput of 1000 cells/s [29], where the spatial resolution of slits was limited by the width of slits, leading to blurry images. In order to address this issue, Lo@UCSD further developed a 3D microfluidic imaging flow cytometry leveraging spatial filters with slits, pinholes and orthogonal light-sheet scanning illumination, enabling the mapping of cell images using calibration beads mixed with cells in 2022 [39] (see Figure 6c). However, this translation of PMT signals into geometrical dimensions of single cells were based on the assumption of previously known cell shapes and cannot be used to image irregular cell organelles such as nucleus of WBC with a variety of geometries.

In 2022, Chen@CAS reported the simultaneous characterization of Single-cell impedance and imaging based on constriction microchannels with a microfabricated metal window. Based on an equivalent bioelectrical model for a cell squeezing through the microchannel, impedance profiles of the whole cell were translated into cell diameter, specific membrane capacitance and cytoplasmic conductivity where fluorescent signals of cell nucleus captured by a PMT were translated into nuclear diameter based on time spatial translation, producing high rates of classifying K562 and Jurkat cells of leukemia [40]. However, in this approach, the nuclear diameter was calculated by the signal detected by PMT where nuclear morphologies were still missing (see Figure 6d).

## 6. Future Directions of Optoelectronic Flow Cytometry

In order to meet the clinical demands of complete blood counting, optoelectronic flow cytometry has witnessed a roughly 70-year development and currently, commercially available instruments of hematology analyzers can classify whole blood samples in an automatic and high-throughput manner and function as the fast-screening approach of analyzing blood samples.

When dealing with normal blood cells, current hematology analyzers can reliably realize 5-part or more differentials of WBC and the differentiation of RBC from PLT. However, excluding intrinsic parameters of cell diameters, different instruments rely on different non-intrinsic parameters for cell-type classification. For instance, in DxH 900 of Beckman Coulter, opacity (a ratio of high-frequency/low-frequency impedance data) is adopted to differentiate RET from RBC, which strongly depends on AC power source of impedance measurements, geometrical structures of apertures as well as electrical differences between RET and RBC.

In addition, although scattered lights have been widely used to differentiate WBC in DxH 900 of Beckman Coulter, XN-1000 of Sysmex, ADVIA 2120i of Siemens and CELL-DYN Ruby of Abbott, the obtained light intensities cannot be effectively compared among different instruments since these optical parameters cannot be translated into intrinsic optical properties of single cells and can be heavily affected by power sources, optical paths and detectors of different instruments. Furthermore, customized chemical treatments are also used in these hematology analyzers such as peroxidase staining in ADVIA 2120i, shrinkage and nuclear staining of non-basophil WBC in XN-1000, which further leads to the quantification of non-inherent biophysical parameters in cell-type classification.

When dealing with abnormal blood cells, since current hematology analyzers based on electrical impedance and optical scattering cannot obtain inherent biophysical parameters of immature blood cells (e.g., cell and nuclear morphologies, cytoplasmic conductivity), they can only function as a fast-screening approach, which is always followed by microscopic smear screening for further examinations of abnormal blood samples.

In order to address these aforementioned issues, recently there are several efforts in developing microfluidic approaches focusing on the characterization of inherent biophysical parameters of single blood cells, with the purpose of producing a well-recognized standard for blood analysis. For instance, intrinsic electrical parameters of blood cells such as membrane capacitance and cytoplasmic conductivity have been measured by microfluidic impedance flow cytometry reported by Chen@CAS [25] and Morgan@Southampton [34], respectively. However, based on these intrinsic bioelectrical parameters (e.g., cell diameter, specific membrane capacitance and cytoplasmic conductivity), only 3-part differential of WBC can be realized while classifications of granulocyte subgroups based on these intrinsic bioelectrical parameters cannot be effectively realized [36,37].

Meanwhile, microfluidic imaging flow cytometry have been recently developed to capture intrinsic geometrical parameters of blood cells such as cell and nuclear morphologies. However, due to the issue of out of focus, high-speed images of travelling single cells have seldom been used to obtain geometrical information and the following cell type classification of WBC. Recently, Chen@CAS reported the development of microfluidic impedance and imaging flow cytometry of capturing both electrical and geometrical parameters of single cells (e.g., cell diameter, nuclear diameter, specific membrane capacitance and cytoplasmic conductivity), producing high classification rates of two leukemia cell lines, although nuclear morphologies were missing [40]. Further developments in this direction are highly demanded where intrinsic biophysical parameters of blood cells can be quantitatively rather than qualitatively measured, and thus, function as the gold standard approach of blood analysis by replacing microscopic smear screening in the near future.

## 7. Conclusions

In this study, historical developments of conventional and microfluidic optoelectronic flow cytometry were reviewed and compared, leading to the following conclusions. Developments of optoelectronic flow cytometry were driven by clinical demands of whole blood counting, which followed the path of demand-driven innovation. Well-established optoelectronic flow cytometry or hematology analyzers can realize 5-part or more differential of normal WBC and trigger an alarm rather than conduct classification when abnormal WBC are encountered. Microfluidic optoelectronic flow cytometry can classify WBC with higher accuracies due to fine-tuned sensitive structures than conventional hematology analyzers and measure a few size-independent intrinsic biophysical markers of single leukocytes. Future directions may focus on the developments of next-generation microfluidic optoelectronic flow cytometry enabling the full characterization of Single-cell intrinsic biophysical parameters to form commonly used quantitative identification systems of leukocytes, leading to the automatic classification of both mature and immature leukocytes.

## Figures and Tables

**Table 1:** A summary of keep developments of hematology analyzers. Reprinted with permission from Ref [16], copyright 2022, Elsevier.

Year	Instrument	Manufacturer	Methodology	Parameter
1950s	Model A	Coulter Electronics	Direct Current (DC) Resistance	
1970s	Model S Plus	Coulter Electronics	DC Resistance	Three-Part Differential of WBC
1980s	Model STKs	Coulter Electronics	DC/AC (Alternating Current) Impedance & Optical Scattering	Five-Part Differential of WBC
1980s	Sysmex NE-8000	TOA Medical Electronics	DC/AC Impedance & Cell Treatment	Five-Part Differential of WBC
1980s	CELL-DYN 3000	Abbott	Multiple-Angle Optical Scattering	Five-Part Differential of WBC
2000s	ADVIA 2120i	Siemens	Multiple-Angle Optical Scattering & Cell Treatment	Five-Part Differential of WBC, NRBC, RET
2010s	DxH 900	Beckman Coulter	DC/AC Impedance & Multiple-Angle Optical Scattering & Cell Treatment	Five-Part Differential of WBC, NRBC, RET
2010s	XN-1000	Sysmex	Multiple-Angle Optical Scattering and Fluorescence & Cell Treatment	Five-Part Differential of WBC, NRBC, RET, IG

**Table 2:** A summary of key developments of microfluidic impedance and imaging flow cytometry.

Year	Group	Methodology	Result	Ref
2001	Renaud@EPFL	Coplanar Microelectrode + Impedance	RBC vs. Ghost Based on AC Impedance	[21]
2005	Renaud@EPFL	Parallel Microelectrode + Impedance	RBC vs. Fixed RBC vs. Ghost Based on AC Impedance	[22]
2009	Morgan@Southampton	Parallel Microelectrode + Impedance	Three-Part Differential of WBC Based on AC Impedance	[23]
2012	Goda@UCLA	Inertial Focusing + PMT	WBC vs. MCF-7, 100,000 cells/s, Differentiation	[24]
2013	Chen@CAS and Sun@Toronto	Constriction Microchannel + Impedance	RBC vs. Neonatal RBC Based on Cell Diameter, Specific Membrane Capacitance and Cytoplasmic Conductivity	[25]
2013	Dao@MIT	Coplanar Microelectrode + Impedance	RBC vs. P. falciparum Infected RBC Based on AC Impedance	[26]
2013	Bashir@UIUC	Coplanar Microelectrode + Impedance	CD4+ and CD8+ LYM Based on AC Impedance	[27]
2014	Morgan@Southampton	Parallel Microelectrode + Optical Waveguide	Three-Part Differential of WBC Based on AC Impedance, Optical Scattering and Fluorescence	[28]
2015	Lo@UCSD	Microfabricated Window + PMT	A549, 1000 cells/s, Imaging	[29]
2017	Bashir@UIUC	Coplanar Microelectrode + Impedance	CD64+ NEU and MONO Based on AC Impedance	[30]
2017	Chen@CAS	Constriction Microchannel + Impedance	GRA vs. LYM Based on Membrane Capacitance and Specific Membrane Capacitance	[31]
2017	deMello@ETH	Inertial Focusing + sCMOS	HL-60, HeLa, Live, Early and Late Apoptotic Jurkat, 50,000 cells/s, Imaging	[32]
2019	Lo@UCSD	3D Microfabricated Window + PMT	HEK-293, CMK3, 500 cells/s, Imaging	[33]
2020	Morgan@Southampton	Parallel Microelectrode + Maxwell’s Mixture Theory	RBC vs. Ghost Based on Cell Diameter, Specific Membrane Capacitance, Cytoplasmic Conductivity and Cytoplasm Permittivity	[34]
2021	deMello@ETH	Viscoelastic Focusing + sCMOS	Yeasts, 293T, B-Lymphoid, Jurkat, 60,000 cells/s, Imaging	[35]
2022	Chen@CAS	Constriction Microchannel + Impedance	Three-Part Differential of WBC Based on Cell Diameter, Specific Membrane Capacitance and Cytoplasmic Conductivity	[36]
2022	Chen@CAS	Constriction Microchannel + Impedance	Five-Part Differential of WBC Based on AC Impedance	[37]
2022	Morgan@Southampton	Parallel Microelectrode + Convolutional Neural Network	RBC vs. Ghost Based on Cell Diameter, Membrane Capacitance, Cytoplasm Conductivity, Cytoplasm Permittivity	[38]
2022	Lo@UCSD	3D Microfabricated Window + PMT	HEK-293, HeLa, MCF-7, MCF-10A, 1000 cells/s, Differentiation	[39]
2022	Chen@CAS	Constriction Microchannel + Microfabricated Window + Impedance + PMT	K562 vs. Jurkat, SACC-LM vs. CAL-27, Differentiation	[40]

## Data Availability

Not applicable.
